# Target-Oriented Reference Construction for supervised cell-type identification in scRNA-seq

**DOI:** 10.21203/rs.3.rs-4559348/v1

**Published:** 2024-06-26

**Authors:** Xin Wei, Wenjing Ma, Zhijin Wu, Hao Wu

**Affiliations:** Brown University; University of Michigan–Ann Arbor; Brown University; Shenzhen University of Advanced Technology

**Keywords:** scRNA-seq, Supervised learning, Cell-type identification, Reference construction

## Abstract

Cell-type identification is the most crucial step in single cell RNA-seq (scRNA-seq) data analysis, for which the supervised cell-type identification method is a desired solution due to the accuracy and efficiency. The performance of such methods is highly dependent on the quality of the reference data. Even though there are many supervised cell-type identification tools, there is no method for selecting and constructing reference data. Here we develop Target-Oriented Reference Construction (TORC), a widely applicable strategy for constructing reference given target dataset in scRNA-seq supervised cell-type identification. TORC alleviates the differences in data distribution and cell-type composition between reference and target. Extensive benchmarks on simulated and real data analyses demonstrate consistent improvements in cell-type identification from TORC. TORC is freely available at https://github.com/weix21/TORC.

## Background

Computational cell-type identification (referred to as “cell-typing” hereafter) is the most fundamental step in single-cell RNA sequencing (scRNA-seq) analysis [1]. Supervised cell-typing has gained increasing popularity over unsupervised clustering, due to better accuracy and robustness [2]. Supervised cell-typing trains on a reference (training) sample of cells with known cell-type labels, and assigns labels to cells in a target (testing) sample using the trained classifier [3]. A key factor that determines a classifier’s success is the quality of the training data, especially the similarity between the reference and the target populations [4]. A natural idea is to expand the sources of the reference and increase the reference size. However, the benefit of a larger reference sample is limited by its quality.

Much effort has been put into developing new cell-typing algorithms [5–7]. Not enough attention is given to selecting and constructing reference data, which we argue is more fundamental to the choice of algorithm. To fill the gap, we develop a novel method named “Target-Oriented Reference Construction (TORC)”. We first demonstrate the importance of reference quality and show that the reference quality should be viewed in respect to the target, thus an appropriate reference is target-oriented. TORC provides a general strategy that minimizes the difference in cell-type composition as well as cell-type-specific expression profiles between the reference and the target. We demonstrate the improvement from using TORC in extensive real data examples.

## Results and Discussion

### Algorithm Overview

**Figure 1.**
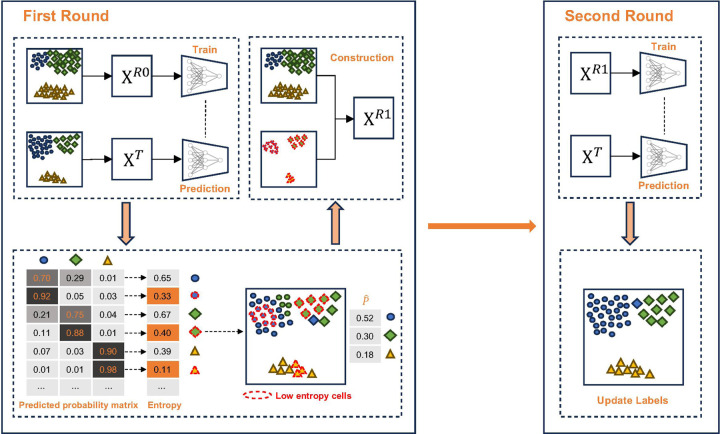
Overview of TORC framework. The main goal of TORC is to construct a reference that closely resembles the target. TORC employs a two-round prediction strategy. In the first round, a classifier is trained on a reference with known cell labels to predict the cell-type composition of the target. Using the predicted probability matrix from this initial prediction, entropies are calculated for each cell. Cells with relatively low entropies are then added to expand the reference pool. A target-oriented reference is subsequently created by sampling from the expanded reference pool according to the estimated cell-type composition. The constructed reference is then used to retrain the classifier and update the cell labels of the target.

TORC aims to construct a reference suitable for a given target data. It first uses an off-the-shelf supervised method to label the target cells, from which TORC first estimates cell-type composition in the target. TORC then add target cells with high-confidence labels to the reference to form an expanded reference pool. Then TORC resamples from the pool to construct a new reference according to the target composition. The reconstructed reference is used to build the final classifier (Fig. 1).

### Study design

A benchmarking of supervised cell-typing for scRNA-seq [8] investigated key factors affecting the performance: feature selection, prediction method, and choice of the reference dataset. They found that multi-layer perceptron (MLP) [9] combined with F-test-based feature selection generally performs the best. Based on these observations, we focus on using MLP to demonstrate the results from the TORC. Results based on ACTINN [10], scNym [11] and scANVI [12] are also included to show the generalizability of TORC. We use Accuracy as the primary assessment metric, which captures the overall percentage of correct cell-type assignments.

### Datasets

All datasets used are listed in Tables S1. We include multiple datasets from human peripheral blood mononuclear cells (PBMC) from 10X sequencing platform. The datasets “Covid CN” [13], “Covid UK” [14], “Covid FMC” [15], “Lupus” [16] and “Protocol” [17] each obtains cells from multiple individuals. The “FACS” dataset [18] includes cells separated by fluorescence-activated cell sorting (FACS). Substantial inter-individual variability due to age, sex, and overall health are present in PBMC, allowing us to examine the impact of different target-reference sample relationships.

#### Reference quality affects accuracy and depends on the target sample

Generally speaking, accuracy increases as reference size grows [8]. However, reference quality is just as important. Adding more cells to the reference can deteriorate the classification. Table S2 shows that, depending on the target, using cells from a single subject as reference can outperform using all 39 subjects from the entire study.

To investigate the reference impact, we compare the performance from three references on 21 targets. Figure S1 shows a two-fold reference effect. First, some references are better in general. Second, a good general reference is not always the best choice for all targets. For example, reference “batch1 1079” has high accuracy in general, but it is not the best for target “control 1016”. Therefore, choosing the best reference has to take the target into consideration.

### Both domain shift and composition difference exist in real data

An implicit assumption in most learning scenarios is that reference and the target follow the same distribution. Thus, in the scRNA-seq context, it is ideal if all training cells are from the same biological source as the target. In reality, deviation between the two populations always occurs. This discrepancy calls for a balance between quantity and quality. The reference quality is attributable to two main aspects: the similarity in the distribution of expression profiles and the similarity in cell-type composition. Figure S2.A shows an example with obvious of domain shift while Figure S2.B shows an example with little domain shift, but with large differences in cell-type composition.

### Constructing a reference that reflects the target cell composition improves accuracy

**Figure 2.**
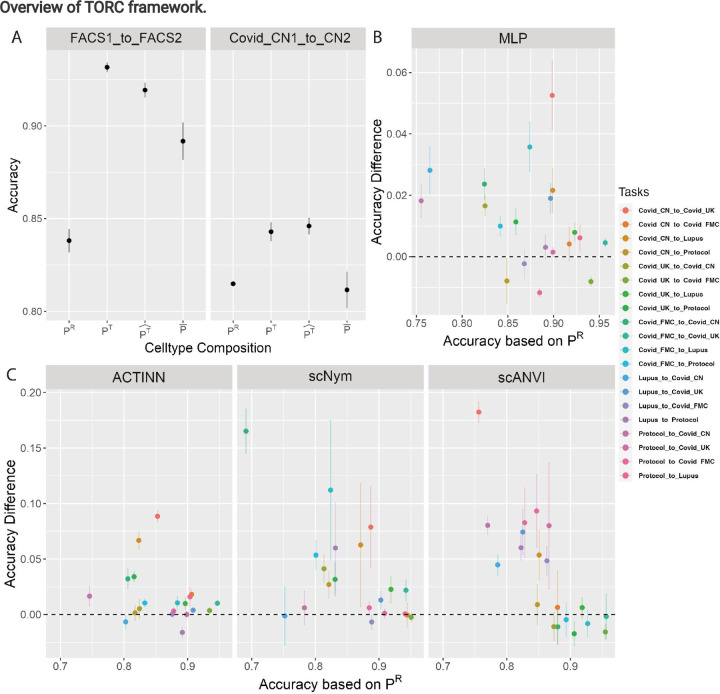
Gain from employing TORC. TORC enhances the predictions on multiple human PBMC datasets. **A** The average accuracy from MLP on 50 different random seeds when using i. original reference with cell-type proportion *P*^*R*^; ii. constructed reference with cell-type proportion *P*^*T*^ which is same as true cell-type composition of target set; iii. constructed reference with cell-type proportion $\hat{P^{T}}$ estimated from first round of TORC; and iv. constructed reference with equal weighted cell-type proportion $\bar{P}$. The vertical bar indicates the standard deviation of the accuracy in the 50 runs. **B** The gain in accuracy using the TORC constructed reference versus using the original reference. Each data point represents the average from MLP on 50 different random seeds for specific analysis task, such as using a reference generated from “Covid CN” to predict the target “Covid UK”. The vertical bar indicates the standard deviation of the accuracy gain in the 50 runs. **C** The gain in accuracy using other cell-typing algorithms (ACTINN, scNym and scANVI) with the same TORC constructed reference as in **B** versus using the original reference.

First, we demonstrate the benefit of constructing a reference that reflects the target cell composition using a toy example. The “FACS” dataset comprises nine subtypes identified through FACS experiments, commonly regarded as the “gold standard” dataset. The cytotoxic T and naive cytotoxic T cells are the most difficult to distinguish (Figure S3.A). We create a scenario where the odds of these two cell-types are reversed between the reference and target, while the proportions of other cell-types remain the same (Table S3). Trained with all cells in the reference, the accuracy is 0.84. However, if cells are sampled from the reference pool according to the target cell composition to form the training set, the accuracy increases sharply to 0.9 (Fig. 2.A). When an estimated target cell composition is used in place of the true composition, the accuracy still shows substantial gain. These improvements are due to the reduction of cytotoxic T cells misclassified as naive cytotoxic T cells (Figure S3.B, Figure S3.C).

The FACS dataset includes cells from only one source. We next assess the performance of TORC when the reference and target include cells from multiple subjects from the same study (Table S4). The improvement brought by the TORC is consistent (Fig. 2.A).

A reusability report of scBERT [19] indicates that the role of cell-type distribution is overlooked and taking a reference with balanced cell-type weights may improve prediction [20]. Though this approach ensures every cell-type is well represented in reference, it does not reflect the target composition. TORC consistently surpasses the approach of equal-weighted sampling (Fig. 2.A), indicating that leveraging a target-oriented $\hat{P^{T}}$ yields greater benefits compared to a uniform, target-blind $\bar{P}$.

### Expanding the reference pool reduces bias due to domain shift

Next, we apply the TORC using three public COVID datasets and two non-COVID datasets as targets to examine the situation where the reference samples are from different studies. Since reference cells are from biological samples in studies different from the target, we employ the reference expansion option in the TORC to include some target cells with low prediction entropy (high confidence) in the expanded reference pool. In most reference-target pairs, using the reference constructed by TORC leads to increased accuracy (Fig. 2.B, Figure S4). Using the same TORC also improves accuracy using ACTINN, scNym and scANVI in most situations (Figure S5).

### Use MLP-based reference

In practical applications, if a researcher has a particular interest in a specific supervised classification method such as scANVI, apart from directly applying TORC with scANVI as the algorithm for both the reference construction and ultimate classification, we provide a flexible, alternative approach. This involves using MLP in reference construction and, once a target-oriented reference is constructed, using the user’s choice of classification method for cell-type identification. Using MLP in reference construction is computationally efficient (Figure S6) and algorithms such as ACTINN, scNym and scANVI benefit from MLP-based reference construction (Fig. 2.C, Figure S7.A, Figure S7.B)

## Discussion

TORC constructs a reference sample with the target in mind to address the common issue of dataset shift in scRNA-seq cell-typing. The essence of the algorithm is to consider the complexity in the relationship between reference and target. We find two major factors that affect the classification accuracy: the cell-type-specific expression profile and cell-type composition.

In this paper, we aim to point out the importance of reference, in addition to the choice of algorithm, in supervised scRNA-seq cell-type identification. We view the current TORC as a beginning and see many potential extensions as public data continues to accumulate. For example, available reference samples can be rated by their quality, reflected by the accuracy in classifying cells in other labeled references. Cells of different types within a reference sample may be associated with different quality and a good construction may sample cells from multiple sources.

## Conclusions

As scRNA-seq becomes increasingly applied, particularly in large-scale population-level studies, cell-type identification remains among the most crucial aspects. Even though there are many supervised cell-typing methods, no work has addressed the problem of selecting reference data. To fill this gap, we propose a widely applicable target-oriented reference reconstruction strategy and validate the effectiveness and practicality of the TORC. Both simulated and real data analyses have showcased the potential of this strategy which points out future research interest and directions. For example, a similar strategy can be applied to other single cell assays such as scATAC-seq and spatial transcriptomics.

## Data Availability

The implementation of the MLP-based TORC is available at https://github.com/weix21/TORC. Several datasets are analyzed in this manuscript, as summarized in Table S1. All datasets analyzed are publicly available in the Gene Expression Omnibus (GEO), UCSC Cell Browser, COVID-19 Cell Atlas, Single Cell Portal (SCP) and 10X Genomics platform with the following accession numbers: “Covid CN” [13] (GSE158055), “Covid UK” [14] (COVID-19 PBMC Ncl-Cambridge-UCL), “Covid FMC” [15] (covid19-immuno), “Lupus” [16] (GSE96583), “Protocol” [17] (SCP424) and “FACS” [18] (10X Genomics Datasets).
